# Full Atrioventricular Block Secondary to Acute Poisoning Mercury: A Case Report

**DOI:** 10.3390/ijerph15040657

**Published:** 2018-04-02

**Authors:** Amelia Geraldine Peregrina-Chávez, María del Rayo Ramírez-Galindo, Rolando Chávez-Martínez, Cesar Anuar Delahanty-Delgado, Fernando Vazquez-Alaniz

**Affiliations:** 1 Urgency Department, Hospital General 450, Servicios de Salud de Durango, Blvd. Jose Maria Patoni No. 403, Col. El Cipres, CP 34206 Durango, Mexico; geraldineperegrina@gmail.com (A.G.P.-C.); draramirezurg@hotmail.com (M.d.R.R.-G.); 2 Cardiology Department, Hospital General 450, Servicios de Salud de Durango, Blvd. Jose Maria Patoni No. 403, Col. El Cipres, CP 34206 Durango, Mexico; dr_chavez81@hotmail.com (R.C.-M.); dr.delah2@gmail.com (C.A.D.-D.); 3 Clinical Laboratory, Hospital General 450, Servicios de Salud de Durango, Blvd. Jose Maria Patoni No. 403, Col. El Cipres, CP 34206 Durango, Mexico

**Keywords:** mercury exposure, atrioventricular block, gold mining

## Abstract

*Background:* The biological behaviour and clinical significance of mercury toxicity vary according to its chemical structure. Mercury differs in its degree of toxicity and in its effects on the nervous, digestive and immune systems as well as on organs such as the lungs, kidneys, skin, eyes and heart. Human exposure occurs mainly through inhalation of elemental mercury vapours during industrial and artisanal processes such as artisanal and small-scale gold mining. *Case presentation:* A 52-years-old female, housewife, with a body mass index of 25.3 kg/cm^2^, without smoking or alcohol habits or any important clinical or chronic cardiovascular history, was admitted to the emergency room due to probable accidental poisoning by butane gas. Clinical manifestations with a headache, dizziness, cough, and dyspnoea of medium to small efforts. An initial physical exploration with Glasgow scored at 15, with arrhythmic heart sounds, pulmonary fields with bilateral subcrepitant rales and right basal predominance. Electrocardiographic findings were as follows: a cardiac frequency of 50 beats per minute and atrioventricular dissociation. Laboratory parameters were: white blood cells at 15.8 × 10^9^/L; aspartate aminotransferase at 38 U/L; lactate dehydrogenase at 1288 U/L; creatine-kinase at 115 U/L; CK-MB fraction at 28 U/L; and other biochemical parameters were within the reference values. A radiographic evaluation showed flow cephalization, diffuse bilateral infiltrates with right basal predominance. In addition, the patient presented data of low secondary expenditure to third-degree atrioventricular (AV) block for which the placement of a transvenous pacemaker was decided, substantially improving the haemodynamic parameters. Subsequently, after a family interrogation, the diagnosis of mercury inhalation poisoning was established. An initial detection of mercury concentration (Hg(0)) was carried out, reporting 243.5 µg/L. In view of this new evidence, mercury chelation therapy with intravenous calcium disodium ethylenediamine tetraacetic acid (CaNa_2_·EDTA) was initiated. After 8-days of hospital stay, she presented a favourable evolution with both clinical and radiological improvements, so that the mechanical ventilation progressed to extubating. Subsequently, she was referred for cardiology because of her persistent 3rd-degree atrioventricular block, deciding to place a definitive bicameral pacemaker. The patient was discharged from the hospital 14 days after admission due to clinical improvements with mercury plasma levels at 5 µmol/L and a heart rhythm from the pacemaker. *Conclusions:* We show evidence that acute exposure to elemental mercury can affect the heart rhythm, including a complete atrioventricular blockage.

## 1. Background

Mercury has three valence states and exists in several forms: inorganic mercury, which includes liquid metallic mercury and mercury vapour Hg(0), mercurous Hg(I) and mercuric Hg(II) salts, and organic mercury with methylmercury (CH_3_Hg, MeHg), ethylmercury (C_2_H_5_Hg, EtHg), and phenylmercury (C_6_H_5_Hg, PhHg). The biological behaviour and clinical significance of the various forms of mercury vary according to its chemical structure [1]. These forms of mercury differ in their degree of toxicity and in their effects on the nervous, digestive and immune systems, as well as on the lungs, kidneys, skin, eyes and heart [2]. Human exposure occurs mainly through the inhalation of elemental mercury vapours during industrial and artisanal processes and through the consumption of contaminated foods. Artisanal and small-scale gold mining (ASGM) is defined in the Minamata Convention on Mercury as “gold mining conducted by individual miners or small enterprises with limited capital investment and production”. ASGM is carried out in over 70 countries by approximately 10–15 million miners, including approximately 4–5 million women and children [3]. ASGM has many associated environmental and occupational health issues, particularly when practiced informally or with limited technical and material resources [4]. The toxicities of different chemical forms of mercury vary greatly. Organic mercury and vapor mercury possess strong toxicity on the central nervous system [5,6]. This fact was demonstrated by Jepson et al. [7], who showed that the concentration of mercury in the gas phase could be up to 35% greater than the levels of pure mercury. Soluble inorganic mercury is characterized by severe renal toxicity and gastrointestinal toxicity [6,8]. The ingestion of liquid metallic mercury or “quicksilver” does not appear to be toxic in itself, and the health hazards from quicksilver are due to its potential to release mercury vapour [5,6]. For decades, the toxic effects of mercury were associated mainly with the central nervous system. However, a growing body of evidence suggests that mercury exposure can also lead to increased risks of adverse cardiovascular impacts in exposed populations [9], mainly by organic and inorganic mercury exposure [10].

## 2. Ethics, Approval and Consent to Participate

This case report was approved for publication by the ethic and investigation committees at Hospital General 450. Written informed consent was obtained from the patient for the publication of this case report. A copy of the written consent is available for review by the editor-in-chief of this journal.

## 3. Case Presentation

**Figure 1:**
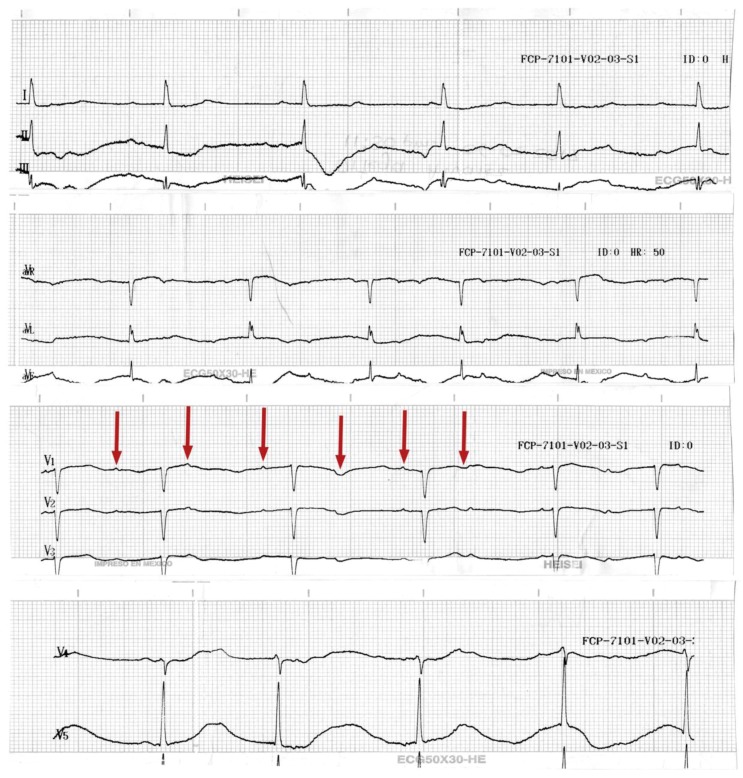
Electrocardiogram of the patient on admission to the emergency room. It shows a dissociation of P waves (V_1_ Red arrows) from the QRS complexes.

**Figure 2:**
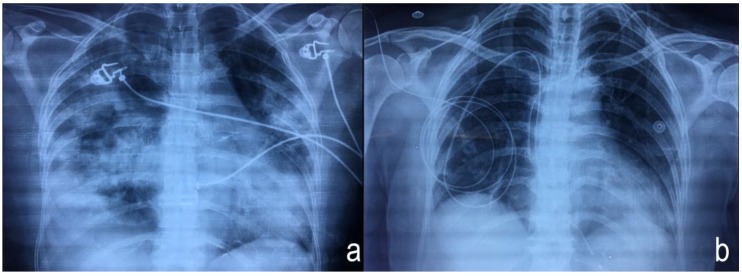
Chest X-ray (**a**) showing diffuse bilateral infiltrates with right basal predominance and chest X-ray (**b**) after chelation showing the transvenous pacemaker.

The patient was a female 52-years-old, housewife, resident of the rural zone in Durango, Mexico. The patient body mass index was 25.3 kg/cm^2^, without smoking or alcohol habits or any important clinical or chronic cardiovascular history. The case was presented by paramedical staff in the emergency room (ER) as a probable accidental poisoning by butane gas. Clinical manifestations begin with a headache, dizziness, cough, and dyspnoea of medium to small efforts. A cardiac frequency of 44 min, arterial tension (AT) at 96/46 mm Hg; an oxygen saturation (O_2_*sat*) of 99%; a fraction of inspired oxygen (FiO_2_) of 40%. The arterial gasometry at a pH of 7.40; pCO_2_ at 35 mmHg; partial pressure of Oxygen (*pa*O_2_) at 94 mm Hg; bicarbonate (HCO_3_) at 21.7 mmol/L; lactic acid (Lactate) at 0.70 mmol/L; and an oxygen saturation (O_2_*sat*) at 97%. The Kirby index (*pa*O_2_/FiO_2_) was 235. An initial physical exploration with Glasgow scored at 15, with arrhythmic heart sounds, pulmonary fields with bilateral subcrepitant rales and right basal predominance. Electrocardiographic findings were: a cardiac frequency of 50 beats per minute (BPM), along with atrioventricular (AV) dissociation (Figure 1). Laboratory parameters were: white blood cells at 15.8 × 10^9^/L; neutrophils at 14.3 mm^3^; aspartate aminotransferase (AST) at 38 U/L; lactate dehydrogenase (LD) at 1288 U/L; creatine-kinase (CK) at 115 U/L; CK-MB fraction (CK-MB) at 28 U/L; and creatinine at 0.8 mg/L; other biochemical parameters were within the reference values. A radiographic evaluation showed flow cephalization, diffuse bilateral infiltrates with right basal predominance (Figure 2a).

Management with non-invasive mechanical ventilation was initiated with continuous positive airway pressure (CPAP) modality following the criteria for a moderate acute respiratory distress syndrome. In addition, the patient presented data showing low secondary expenditure to third-degree AV block (AVB), documented by the electrocardiogram (EKG) above, for which the placement of a transvenous pacemaker was decided, substantially improving the haemodynamic parameters.

Subsequently, she was admitted to the intensive care unit (UCI) and diagnosed with chemical pneumonitis/moderate acute respiratory distress syndrome (ARDS)/poisoning by butane gas/full AVB post colocation temporal pacemaker. Twenty-hour hours after admission in UCI, she showed a worsening respiratory pattern, with a Kirby score of 138 and an ARDS progressing from moderate to severe, requiring advanced respiratory management. After 5-days the patient showed no clinical improvement and presented a torpid progression and non-compatible symptoms with the presumptive diagnosis. Posteriorly, this led to an exhaustive interrogation with a family member who referred to a possible exposure to elementary liquid mercury by inhalation resulting from exposure to mercury used inside the home to extract small-scale gold and to the reported death, some days ago, of 4 persons (54-year-old male, 23-year-old pregnant women with a 32-weeks gestation period, and two children: a 3 year old male and a 1.5 year old female) who were members of the same family and who suffered the same exposure. Regarding this new history, a blood sample was take in a specific tube (BD Hemogard™/Royal Blue with K_2_EDTA 10.8 mg) for the determination of trace elements. An initial blood mercury (Hg(0)) quantitation was carried out via inductively coupled plasma-mass spectrophotometry (ICP-MS) at a reference laboratory, reporting a concentration of 243.5 µg/L (reference to free exposure <20 µg/L). In agreement with Environmental Protection Agency (EPA) recommendations, the values of total blood mercury among women in the 95th percentile remain between 3.7 and 4.5 μg/L [11]. In view of this new evidence, mercury chelation therapy with intravenous calcium disodium ethylenediamine tetraacetic acid (CaNa_2_·EDTA) was initiated with a dosage of 500 mg/m^3^/3 h for two consecutive days. Likewise, two ampules of calcium gluconate at 10% and 5 g of C vitamin were administered simultaneously with the CaNa_2_·EDTA. 

**Figure 3:**
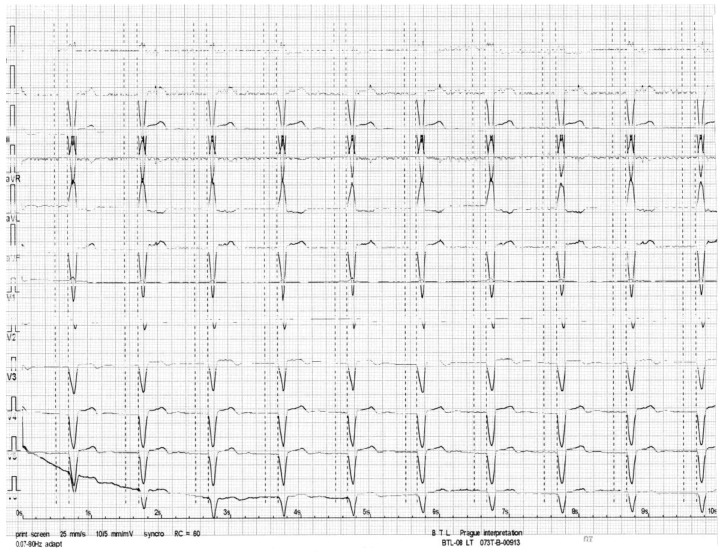
Electrocardiogram showing the stimulation of the bicameral pacemaker after definitive implanting.

After 8-days of hospital stay, the patient presented a favourable evolution with a clinical and radiological improvement that led to the mechanical ventilation progressing to extubating (Figure 2b). Subsequently, she was referred for cardiology because of her persistent 3rd-degree AVB, deciding to place a definitive bicameral pacemaker. The patient was discharged from hospital 14 days after admission due to clinical improvements with mercury plasma levels of 5 µg/L and a heart rhythm from the pacemaker (Figure 3).

## 4. Discussion and Literature Revision

At the Minamata convention in 2013, 128 countries signed up to accept the use of mercury-free products and limit mercury emissions, recognizing that, “Mercury, is a chemical that worries the world because of its long-range atmospheric transport, its persistence in the environment once it has been anthropogenically introduced, its ability to bioaccumulate in ecosystems, and its significant negative effects on human health and the environment” [12]. On the other hand, The International Labour Organization describes artisanal and small-scale mining as “… labour intensive, with mechanization being at a low level and basic”. Building on this description, the World Bank’s Communities, Artisanal and Small-Scale Mining (CASM) initiative elaborates on the economic and social effects of CASM work as being largely “a poverty-driven activity, typically practiced in the poorest and most remote rural areas of a country by a largely itinerant, poorly educated populace with little other employment alternatives” [13].

First, to assess mercury poisoning in our patient, we decided to quantify the serum mercury concentration with ICP-MS technology because this is the method currently used to evaluate toxic-metal levels in blood and/or urine samples in humans [14,15,16].

The first management of the patient in the ER was to resolve ARDS with non-invasive mechanical ventilation due to probable poisoning by methane gas. Once the cause of the poisoning was established, we knew that acute elemental mercury poisoning by inhalation presents a symptomatology related to respiratory insufficiency, dyspnoea, dry coughing, fever and chills, evolving to interstitial pneumonitis with evidence of atelectasis and emphysema leading to the development of ARDS, (characteristics presented in our patient, as shown in Figure 2a), inducing mitochondrial dysfunction with reduced adenosine triphosphate (ATP), the depletion of glutathione and increased lipid peroxidation [17].

Mercury links to numerous biological structures blocking their activity. Indeed, it has a high affinity for sulfhydryl groups (-SH) of aminoacids, proteins, enzymes, and sulfur-containing antioxidants such as *N*-acetylcysteine (NAC), α-lipoic acid (ALA), and glutathione (GSH). Glutathione provides about 30–40% of the plasma antioxidant capacity and is the most potent intracellular and mitochondrial antioxidant for protecting against oxidative stress, inflammation, and cardiovascular disease [9]. Other toxic effects are related with different cellular mechanisms, such as; the interruption of microtubule formation, the changing of the intracellular calcium balance and the membrane potential, the alteration of cell membrane integrity, the disturbing or inhibition of enzymes, the inducing of oxidative stress, the inhibition of protein and DNA synthesis, and the disturbing of immune functions [18]. The vascular effects of mercury include increases in oxidative stress and inflammation [19], reductions in oxidative defenses [20], thrombosis [21], mitochondrial dysfunction [22], depolarization, autoxidation of the inner infarction [10], and cardiovascular disease [9,23]. Mercury toxicity is indeed strongly correlated with hypertension, coronary heart disease, myocardial infarction, cardiac arrhythmias, carotid artery obstruction, cerebrovascular accidents, and generalized atherosclerosis [9,24]. In animal models exposed to inhaled mercury vapour, the cardiovascular and cardiac pathology includes bradycardia, thrombosis in small and medium caliber arteries, focal necrosis with thickening of the endocardium of the perivalvular regions, papillary muscles and valves, endothelial proliferation with inflammatory foci and focal oedema, inflammation, and fibrosis of the ascending aorta [23].

Heavy metals can antagonize Ca^2+^ at the actin–myosin junction in a concentration-dependent way, producing a progressive decline of the sarcomere contraction and thus of myocyte function. On pharmacologic grounds, drugs such as the inotropic agents that work on increasing intracellular Ca^2+^ may find the reason of their inefficacy to be dilated cardiomyopathy [22]. The clinical and cardiologic presentation of our patient was a complete ventricular atrial blockage, which was evidenced by the electrocardiographic trace; due to the absence of a clinical history related to some chronic cardiovascular event in the past, the atrioventricular blockage was attributed to mercury poisoning. There are controversies regarding the association of coronary events related to mercury exposure. For example, Zhang et al. [25] identified a strong positive association between blood Hg and MeHg and total cholesterol in adolescents in an adjusted model, but no associations with other cardiovascular disease (CVD) risk factors. However, Houston et al. found, in the overall population, several reports describing a close link between mercury and cardiovascular diseases such as carotid atherosclerosis, myocardial infarction, coronary heart disease and hypertension [23]. In this respect. Wildeman et al. [10] observed that Hg(II) affects the cardiovascular systems, and they report a prolonged PR interval, mainly by inorganic Hg(II) mercury exposure, as well as showing a significant prolongation of the QRS and QT intervals, suggesting that the electrical activity of the heart might be more susceptible to Hg(II). In his study, the QRS interval, which represents the depolarization of the ventricles, was most sensitive to combined metal exposure. A prolonged QRS or QT interval is associated with increased risk of arrhythmias and sudden cardiac death [26,27]. Ratios based on reference values show a significant prolongation of the QRS and QT intervals. The main difference between regulatory and environmental ratios is the higher amount of Hg(II) in the regulatory ratios. This suggests that the electrical activity of the heart might be more susceptible to Hg(II). In this case report, the exposure and contact presented severe to high concentrations.

On the other hand, Mozaffarian et al. [28] found no evidence of any clinically relevant adverse effects of mercury exposure on coronary heart disease, strokes, or total cardiovascular disease in U.S. adults at the exposure levels from two cohorts that included 3427 participants.

Optimal management of poisoning by heavy metals is chelation. Chelating agents vary in their specificity for toxic metals [29]. Ideal chelating agents should be: (1) water-soluble, (2) resistant to biotransformation, (3) able to reach sites of metal storage without causing any side effects, and (4) capable of forming nontoxic complexes with toxic metals and of being excreted from the body; and (5) they should also have a low affinity for essential metals, particularly calcium and zinc. Several chelating agents are available, having different affinities for different metals.

In acute mercury intoxication, after metal absorption into the circulatory system, to avoid further distribution and penetration in tissues, the elimination of mercury from the body should be envisaged in order to reduce more serious damage. Employing mercury-chelating agents, inducing diuresis, modulating the urinary pH for metal excretion, employing complexing agents to enhance faecal excretion for metals undergoing extensive enterohepatic circulation, and finally haemodialysis, may all be employed. The applicability and efficacy of these techniques vary depending on the type, intensity, and extent of the exposure and conditions of the patient [9]. A common chelating agent is CaNa_2_·EDTA [30]. It is the most commonly used chelating agent in poisoning lead and cadmium [15]. However, this drug was selected because, in addition to forming complexes that allow the elimination of mercury, several benefits have been reported on the vascular and cardiac systems [31]. In addition, another theory suggests that EDTA therapy may reduce the oxidative stress injury and inflammation in blood vessel walls [32], although these benefits are controversial and not convincing [33].

## 5. Conclusions

Animal model experiments and human studies with chronic mercury exposure have shown evidence of toxic cardiovascular effects. However, in this specific case, we showed evidence that acute exposure to elemental mercury can affect the heart rhythm, including a complete atrioventricular blockage, which must be approached through heart rhythm devices.

## References

[1] Bernhoft R.A. (2012). Mercury toxicity and treatment: A review of the literature. J. Environ. Public Health.

[2] World Health Organization (2008). Exposure to Mercury: A Major Public Health Concern, Preventing Disease through Healthy Environment.

[3] United Nations Industrial Development Organization (2007). Global Mercury Project: Global Impacts of Mercury Supply and Demand in Small-Scale Gold Mining.

[4] World Health Organization (2016). Technical Paper #1: Environmental and Occupational Health Hazards Associated with Artisanal and Small-Scale Gold Mining.

[5] Risher J.F., Concise International Chemical Assessment (2003). Elemental Mercury and Inorganic Mercury Compounds: Human Health Aspects.

[6] Clarkson T.W., Magos L. (2006). The toxicology of mercury and its chemical compounds. Crit. Rev. Toxicol..

[7] Jepson W., Richardson M., Rowlinson J. (1957). The solubility of mercury in gases at moderate pressures. Trans. Faraday Soc..

[8] Liu J., Shi J.Z., Yu L.M., Goyer R.A., Waalkes M.P. (2008). Mercury in traditional medicines: Is cinnabar toxicologically similar to common mercurials?. Exp. Biol. Med..

[9] Genchi G., Sinicropi M.S., Carocci A., Lauria G., Catalano A. (2017). Mercury exposure and heart diseases. Int. J. Environ. Res. Public Health.

[10] Wildemann T.M., Weber L.P., Siciliano S.D. (2015). Combined exposure to lead, inorganic mercury and methylmercury shows deviation from additivity for cardiovascular toxicity in rats. J. Appl. Toxicol..

[11] United States Environmental Protection Agency (2017). America’s Children and the Environment (ACE).

[12] United Nations Environment Minamata Convention on Mercury. http://www.mercuryconvention.org/Convention/tabid/3426/language/en-US/Default.aspx.

[13] International Labour Organization (1999). Social and Labour Issues in Small-Scale Mines.

[14] Liang C., Li Z., Xia X., Wang Q., Tao R., Tao Y., Xiang H., Tong S., Tao F. (2017). Determine multiple elements simultaneously in the sera of umbilical cord blood samples-a very simple method. Biol. Trace Elem. Res..

[15] Ferrero M.E. (2016). Rationale for the successful management of edta chelation therapy in human burden by toxic metals. Biomed. Res. Int..

[16] Delafiori J., Ring G., Furey A. (2016). Clinical applications of HPLC-ICP-MS element speciation: A review. Talanta.

[17] Dias D., Bessa J., Guimaraes S., Soares M.E., Bastos Mde L., Teixeira H.M. (2016). Inorganic mercury intoxication: A case report. Forensic Sci. Int..

[18] Rafati-Rahimzadeh M., Kazemi S., Moghadamnia A.A. (2014). Current approaches of the management of mercury poisoning: Need of the hour. Daru.

[19] Wiggers G.A., Furieri L.B., Briones A.M., Avendano M.S., Pecanha F.M., Vassallo D.V., Salaices M., Alonso M.J. (2016). Cerebrovascular endothelial dysfunction induced by mercury exposure at low concentrations. Neurotoxicology.

[20] Karimi R., Vacchi-Suzzi C., Meliker J.R. (2016). Mercury exposure and a shift toward oxidative stress in avid seafood consumers. Environ. Res..

[21] Lim K.M., Kim S., Noh J.Y., Kim K., Jang W.H., Bae O.N., Chung S.M., Chung J.H. (2010). Low-level mercury can enhance procoagulant activity of erythrocytes: A new contributing factor for mercury-related thrombotic disease. Environ. Health Perspect..

[22] Frustaci A., Magnavita N., Chimenti C., Caldarulo M., Sabbioni E., Pietra R., Cellini C., Possati G.F., Maseri A. (1999). Marked elevation of myocardial trace elements in idiopathic dilated cardiomyopathy compared with secondary cardiac dysfunction. J. Am. Coll. Cardiol..

[23] Houston M.C. (2011). Role of mercury toxicity in hypertension, cardiovascular disease, and stroke. J. Clin. Hypertens..

[24] Solenkova N.V., Newman J.D., Berger J.S., Thurston G., Hochman J.S., Lamas G.A. (2014). Metal pollutants and cardiovascular disease: Mechanisms and consequences of exposure. Am. Heart J..

[25] Zhang Y., Xu C., Fu Z., Shu Y., Zhang J., Lu C., Mo X. (2018). Associations between total mercury and methyl mercury exposure and cardiovascular risk factors in us adolescents. Environ. Sci. Pollut. Res. Int..

[26] Okin P.M., Devereux R.B., Howard B.V., Fabsitz R.R., Lee E.T., Welty T.K. (2000). Assessment of QT interval and QT dispersion for prediction of all-cause and cardiovascular mortality in American Indians: The strong heart study. Circulation.

[27] Zulqarnain M.A., Qureshi W.T., O’Neal W.T., Shah A.J., Soliman E.Z. (2015). Risk of mortality associated with QT and JT intervals at different levels of QRS duration (from the third national health and nutrition examination survey). Am. J. Cardiol..

[28] Mozaffarian D., Shi P., Morris J.S., Spiegelman D., Grandjean P., Siscovick D.S., Willett W.C., Rimm E.B. (2011). Mercury exposure and risk of cardiovascular disease in two U.S. Cohorts. N. Engl. J. Med..

[29] Smith S.W. (2013). The role of chelation in the treatment of other metal poisonings. J. Med. Toxicol..

[30] Sharma B., Singh S., Siddiqi N.J. (2014). Biomedical implications of heavy metals induced imbalances in redox systems. Biomed. Res. Int..

[31] Quan H., Ghali W.A., Verhoef M.J., Norris C.M., Galbraith P.D., Knudtson M.L. (2001). Use of chelation therapy after coronary angiography. Am. J. Med..

[32] Miller K.L., Liebowitz R.S., Newby L.K. (2004). Complementary and alternative medicine in cardiovascular disease: A review of biologically based approaches. Am. Heart J..

[33] Flora S.J., Pachauri V. (2010). Chelation in metal intoxication. Int. J. Environ. Res. Public Health.

